# Metabolic Study of Cucumber Seeds and Seedlings in the Light of the New, Controversial Trend of Preventive Use of Systemic Fungicides

**DOI:** 10.3390/ijms24065554

**Published:** 2023-03-14

**Authors:** Anna Kafka, Dorota Wieczorek, Beata Żyszka-Haberecht, Jacek Lipok

**Affiliations:** Department of Pharmacy and Ecological Chemistry, Institute of Chemistry, University of Opole, Oleska 48, 45-052 Opole, Poland; anna.kafka@uni.opole.pl (A.K.); beata.zyszkahaberecht@uni.opole.pl (B.Ż.-H.); jacek.lipok@uni.opole.pl (J.L.)

**Keywords:** fungicides, plant metabolism, cucumber, *Cucumis sativus*

## Abstract

Cucumber is one of the most commonly produced vegetable crops. The greatest economic losses in the yields of these crops have resulted from fungal infections—powdery mildew and downy mildew. The action of fungicides not only affects the fungi, but can also lead to metabolic disorders in plants. However, some fungicides have been reported to have positive physiological effects. Our research focused on the action of two commercially available fungicides, Scorpion 325 SC and Magnicur Finito 687,5 SC, on plant metabolism. Two approaches were used to check the effect of the fungicides at the early stage of plant development when metabolic changes occur most dynamically: spraying on the leaves of cucumber seedlings and presowing seed treatment. The application of the fungicide formulation as a presowing seed treatment caused perturbations in the phytase activity, leading to disorders in the energetic status of the germinating seeds. In addition, the tested preparations changed the morphology of the germinating seeds, limiting the growth of the stem. Furthermore, the application of the tested fungicides on seedlings also showed a disruption in the energetic status and in the antioxidative system. Therefore, the use of pesticides as agents causes a “green effect” and requires a much deeper understanding of plant metabolism.

## 1. Introduction

Cucumber, belonging to the Cucurbitaceae family, is the third most commonly produced fruit vegetable crop in the world. According to Statista.com data, in 2019 and 2020, world cucumber production was over 87 and 91 million metric tons, respectively [1]. Due to the high economic importance of cucumber, many studies have investigated the influence of external factors, such as solid properties [2,3], salinity [4], or water stress [5], on its metabolism and the quality and quantity of the crop. Several causes lead to potential damage in cucumber cultivation. One of the most dangerous environmental factors in cucumber production is fungal infection, especially at the early stage of plant growth.

Downy mildew (caused by the oomycete pathogen *Pseudoperonospora cubensis* [6]) and powdery mildew (caused mainly by *Podosphaera fusca* [7]) are common diseases found in field- and glasshouse-grown vegetables, including cucumber. These fungal pests are mainly transferred by wind, splashing rain [8], or insects [9]; moreover, some reports have indicated that *P. cubensis* can be transmitted by seeds [10]. The impairment of cotyledons and leaves by downy or powdery mildew leads to metabolic changes in these plant parts. Disruptions in processes such as respiration [6], transpiration, and photosynthesis contribute to leaf dieback [11], resulting in reduced crop yield and economic losses. The management of mildew diseases is based on applying mycoparasite-based or more commonly used chemical plant protection products. Biological agents counteracting powdery mildew contain natural antagonistic microorganisms, most often bacteria, such as *Bacillus* (*B. subtilis*, *B. amyloliquefaciens*, *B. pumilus*), but also filamentous fungi (*Gliocladium catenulatum*) [12]. Most of the common fungicides against powdery and downy mildew are based on chemical compounds, such as strobilurins (e.g., azoxystrobin), phenylamides (metalaxyl), benzamides (fluopicolide), carboxylic acid amides (dimethomorph), carbamates (propamocarb), triazole (difenoconazole), and cyanoacetamide-oxime (cymoxanil) [13,14].

Some data indicate that among these different types of pesticides, there are groups of compounds that have an influence not only on the development of pathogenic fungi, but also on plant metabolism. Negative or beneficial physiological and biochemical changes in plants depend on the chemical structure of the applied fungicides. These changes are mostly correlated with enzyme activity or even gene expression and lead to disruption in carbon or nitrogen metabolism and photosynthesis efficiency, therefore influencing the growth and development of plants [15,16]. One example is propamocarb, which causes an increase in the activity of detoxification enzymes (glutathione S-transferase (E.C. 2.5.1.18), glutathione reductase (E.C. 1.6.4.2)) [17,18], the activation of protective enzymes (peroxidase, superoxide dismutase (E.C. 1.15.1.1), catalase) [19], and other defence mechanisms (e.g., lignin production) [20]. Triazole and strobilurin fungicides also possess similar activity; however, in addition to increasing the activity of antioxidant enzymes (such as catalase, superoxide dismutase, and ascorbate peroxidase) [21], these chemicals transiently increase the content of abscisic acid and reduce ethylene production. In addition, influences of these substances on hormonal status have also been reported. Strobilurin fungicides increase the synthesis of cytokinin and auxin [22,23], while triazoles inhibit gibberellin synthesis, thus ensuring better hormonal balance [24,25]. The influence of fungicides on hormonal status may be reflected in plant morphology, such as a reduction in shoot elongation and the stimulation of rooting [25,26]. Plants treated with triazoles have shown an increase in photosynthetic pigments, improved plant growth, and a higher biomass of many root crops [25]. The positive effect on photosynthesis was also determined for strobilurin fungicides by increasing the net rate of photosynthesis through temporary interaction in mitochondria transpiration, as well as the increase in the chlorophyll content, area of green leaves, and delayed leaf senescence [27].

Numerous positive effects of active compounds with fungicidal activity are being considered to improve the quality and yield of crops [28]. Currently, there are some reports about a new trend involving the application of fungicide compounds to healthy organisms as promoting agents. This idea is thought to be controversial, as fungicides (especially systemic ones) are known to act toxically to some aspects of plant metabolism. The extended use of these substances requires a detailed understanding of the effect of their activity on plant metabolism and development. Despite a large amount of information on the effects of individual active chemicals, there is still unsatisfactory information on the effects of market-available preparations of pesticides, which obviously contain more than one active substance. Thus, the aim of the present study was (i) to explore the influences of the application of two commercially available systemic fungicides, Scorpion 325 SC and Magnicur Finito 687,5 SC, on the germination of cucumber seeds and chosen aspects of plant physiology, as well as (ii) to verify whether this new, controversial trend is justified in regard to the metabolic activity of cucumber seeds and seedlings.

## 2. Results

Because the germination of seeds is the most critical time in a plant’s life and germinated seeds are the most vulnerable to negative environmental conditions [29,30], the presentation of our results commences from these early stages of plant development.

### 2.1. Variant 1—Presowing Treatment

#### 2.1.1. Effect of Fungicides on the Germination of Cucumber Plants

**Figure 1:**
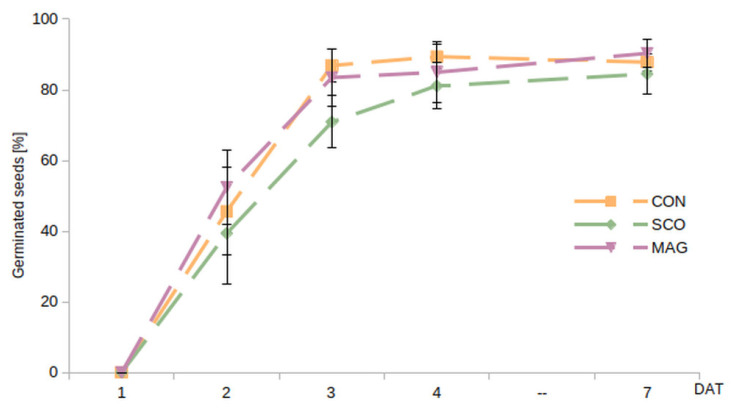
Dynamics of the germination of cucumber seeds given in percentage as the relation of entire number of tested seeds, described as the germination rate. The value differed significantly between SCO and CON, as well as between SCO and MAG, at 3 DAT.

The germination rate (Figure 1) was determined as the percentage of germinated seeds up to 7 DAT (day after seed treatment). A seed was considered to be germinated if the radicle emerged. The radicle was noticeable from 2 DAT, and the highest percentage of germinated seeds was recorded for Magnicur Finito 687,5 SC (MAG) at 52.4%, while the lowest was recorded for Scorpion 325 SC (SCO) at 39.4%. The percentage of seeds germinated reached a maximum value at 4 DAT for the control (CON) and MAG. In contrast, SCO minimally slowed the germination process, but it levelled out at 7 DAT. The highest, but not statistically important, differences were noticed at 3 DAT, in which the control was germinated at 86.9 ± 4.7% and the SCO at 70.9 ± 7.4%.

The development of the germinating cucumber seeds treated with SCO was also slowed. Morphological differences were observed as the limitation of the length of the stem, and a lower number of germinating seeds with green cotyledons were found at 7 DAT compared to CON and MAG.

#### 2.1.2. Effect of Fungicides on Phytase Activity, Phytic Acid, Pi, and Protein Content in Germinating Cucumber Seed Extracts

**Figure 2:**
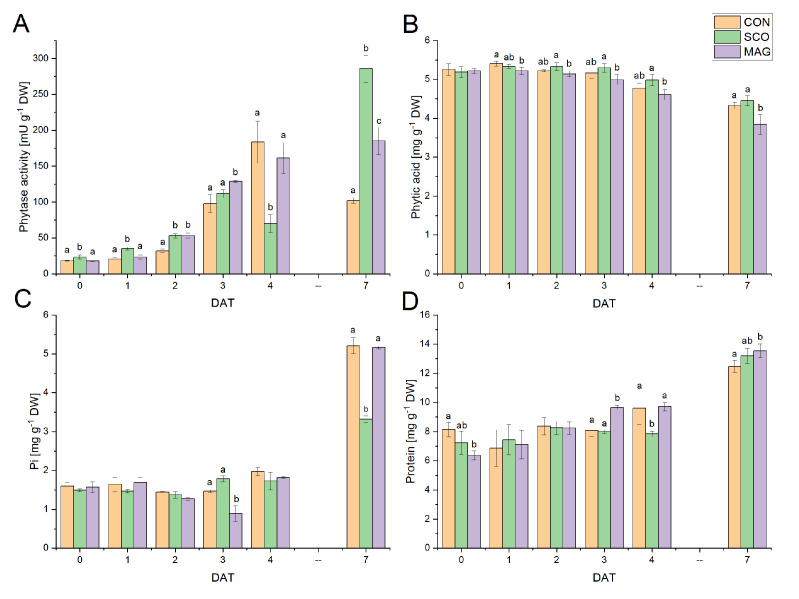
Changes in phytase activity (**A**) and the contents of phytic acid (**B**), orthophosphate (**C**), and soluble protein (**D**) in germinating seeds (up to second day) and whole seedlings of cucumber (3–7 DAT). The presented data involve the presowing seed treatment with fungicides. Different letters represent values significantly different (*p* ≤ 0.05) between treatments for a particular day of development of the germinating seeds.

To verify whether the chosen fungicides affect the germination process, the activities of acid phytase as well as the changes in the contents of phytic acid and Pi were examined (Figure 2). Phytases catalyse the hydrolysis of the ester bond in phytic acid, releasing orthophosphate ions. Acid phytase activity (Figure 2A) increased during germination and reached its maximum value at 7 DAT for SCO (285. 51 ± 18.88) and MAG (184.94 ± 19.20). After 4 DAT, the decrease in enzymatic activity for the control was noticeable and reached a value of 102.19 ± 4.17 mU g^−1^ DW. The content of phytic acid (Figure 2B) ranged from 3.58 to 5.46 mg g^−1^ DW. The lowest value was determined for MAG at 7 DAT, while the highest value was determined for the control at 1 DAT. The significant differences were 1 DAT and 7 DAT between the control and MAG, whereas between the applied fungicides, it was significant from 2 DAT. The content of Pi (Figure 2C) was in the range of 1.23 to 5.41. The Pi content up to 4 DAT was stable, while at 7 DAT, the dynamic increase was noticeable. The changes in phytic acid and Pi did not correlate in a simple way with phytase activities. However, the observed increase in phytase activity could be related to the increase in the protein content (Figure 2D) at the time of germination. The lowest value for the protein content was 6.28 mg g^−1^ DW at 1 DAT for MAG, whereas the highest was 13.55 mg g^−1^ DW at 7 DAT for MAG.

#### 2.1.3. Effect of Scorpion and Magnicur Finito on Adenylate Content and AEC Ratio in Extracts of Germinating Cucumber Seeds

**Figure 3:**
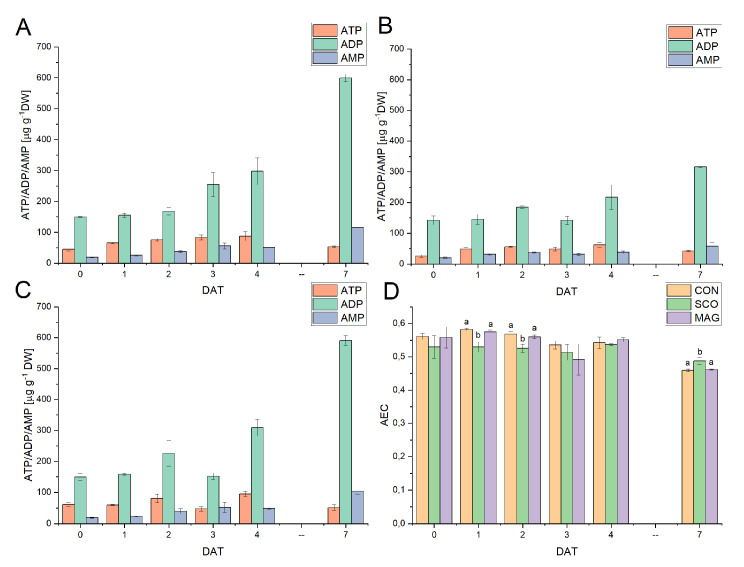
Energetic status of germinating cucumber seeds in control (**A**) or presowing seeds treated with tested pesticides (SCO (**B**) and MAG (**C**)) presented as the content of ATP, ADP, and AMP. Panel (**D**) shows the calculated adenylate energy charge (AEC) in germinating cucumber seeds. Different letters represent values significantly different (*p* ≤ 0.05) between treatments for a particular day of development of the germinating seeds.

Figure 3 presents the influence of fungicides applied as an incrustation agent on the metabolic status of germinated seeds. Changes in the ATP, ADP, and AMP contents for the control (Figure 3A) tended to increase to 7 DAT and were in the ranges of 40.18–103.02 µg g^−1^ DW, 148.04–611.42 µg g^−1^ DW, and 18.50–124.49 µg g^−1^ DW, respectively. However, the exception was ATP, in which the content increased to 4 DAT and decreased to 53.32 ± 2.29 at 7 DAT. With regard to MAG (Figure 3C), a similar upward trend was observed for the content of the three adenylates. In the case of SCO (Figure 3B), the ADP content increased (with an unexpected decrease at 3 DAT), while the ATP and AMP contents fluctuated during the experiment. The value differed significantly from the controls for SCO concerning ATP, which was at 3–7 DAT for SCO and at 2–3 DAT for MAG with respect to ADP, and was 1, 3, 4, and 7 DAT for SCO with respect to AMP.

The AEC ratios (Figure 3D) were in the range of 0.58–0.46, which suggests that anabolic processes predominated. The highest value was observed at 1 DAT, while the lowest was at 7 DAT, both for CON and MAG. For SCO, the AEC ratio was in the range of 0.49 ± 0.01 at 7 DAT to 0.54 ± 0.00 at 4 DAT. In the case of CON and MAG, a decrease in AEC was noticeable, while for SCO, the value fluctuated.

#### 2.1.4. Effect of Fungicides on ^31^P NMR Profiles in Acidic Extracts of Germinating Cucumber Seeds

^31^P NMR was used to characterise all P species, both organic and inorganic phosphorus compounds, in the cucumber seedling extracts. The groups were defined based on characteristic chemical shifts at alkaline pH [31]. For all ^31^P NMR spectra, the peaks lay between −0.2 and 7.0 ppm. The ranges of chemical shifts were set at 5.5–7.0 for orthophosphates, 3.0–5.0 with respect to orthophosphate monoesters, 1.5–3.0 for phospholipids, and (−2)–1.5 ppm for other phosphodiesters. Table 1 presents the phosphorus profiles obtained from the germinated seed extracts at 0, 2, and 4 DAT. The largest share is phosphate monoesters, and their content ranges were from 49.7 to 75.3%. The lowest value was recorded at 4 DAT for MAG, while the highest was in the case of CON at 2 DAT. The share of orthophosphates ranged from 24.5 to 49.8% and correlated with the share of phosphomonoesters; the highest value was recorded for MAG at 4 DAT, and the lowest value was recorded for CON at 2 DAT. On the other hand, the content of phosphodiesters, including phospholipids, ranged from 0.1 to 1.4%. These differences are quantitative. In the SCO, the shares of the individual P groups remained stable, and there were no differences between the days. In the case of MAG, there was an increase in the share of orthophosphates and a decrease in phosphorus monoesters, and at 4 DAT, the share of these groups equalised, reaching values of 49.8% and 49.7%, respectively. In the case of the control, the orthophosphate content initially decreased from 31.5% to 24.5% and then increased to 45.2%.

**Figure 4:**
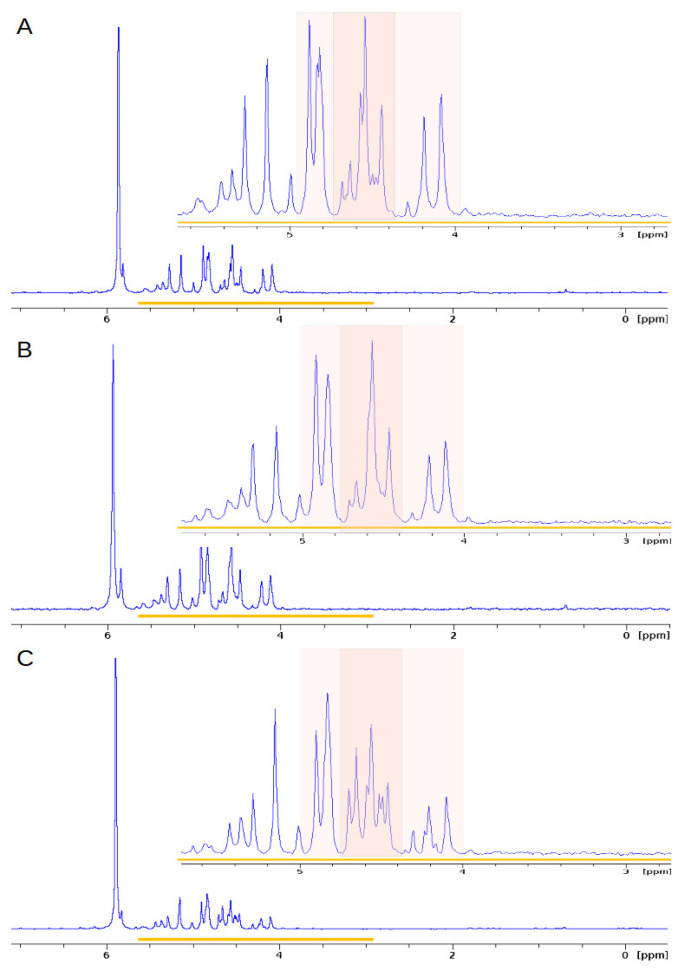
Normalised ^31^P NMR spectra of extracts obtained from germinating seeds treated (as presowing application) with Magnicur Finito 687,5 at 0 (**A**), 2 (**B**), and 4 (**C**) DAT. In the right upper part of the panel, the enlarged part of the spectrum is presented to show subtle differences in its structure. The area watermarked in colour covers the peaks reflected the most important changes.

Based on the data presented graphically as collated spectra in Figure 4, we conclude that the phosphorus profiles changed during the process of germination. The largest differences were noticed in the number of signals located between 4.4 and 5.0 ppm, which correspond to phosphomonoesters. These differences resulted from dynamically progressing biochemical changes—mainly the phosphorylation of compounds involved in metabolic pathways.

### 2.2. Variant 2—Foliar Application

Due to the confirmed information [25,27], the increase in photosynthetic pigments was one of the effects of the Scorpion 325 SC active ingredients (azoxystrobin and difenoconazole) on the plants. Therefore, we decided to present the results starting from this point of view.

#### 2.2.1. Effect of Fungicides on Photosynthetic Pigments in Cucumber Plants

The contents of photosynthetic pigments in the aboveground parts of the cucumber seedlings (shoot includes two green cotyledons and true leaves) are presented in Table 2. The total chlorophyll concentration was in the range of 7.36–10.69 mg g^−1^ DW. The maximum increase in chlorophyll was found at 2 DAT (days after seedling treatment) for all experimental treatments. These changes in the values correspond with the changes in the chlorophyll ‘a’ content. However, the content of chlorophyll ‘b’ was at the same level during the time of the experiment, which was in the range of 2.00–2.70 mg g^−1^ DW. The highest value of chlorophyll ‘b’ was observed at 2 DAT and amounted to 2.38 ± 0.28 for the control (CON), 2.32 ± 0.31 for Scorpion 325 SC (SCO), and 2.50 ± 0.20 for Magnicur Finito 687,5 SC (MAG). The changes in carotenoids in the cucumber seedlings were similar to those in chlorophyll ‘b’, and the values were within the range of 0.61–0.95 mg g^−1^ DW. After 2 DAT, a decrease in the photosynthetic pigment content was observed.

The collected experimental data show that only significant differences in the concentration of photosynthetic pigments were observed after 24 h between CON and SCO with respect to chlorophyll ‘a’, SCO and MAG with respect to chlorophyll ‘b’, and between the control and applied fungicides with respect to the concentration of carotenoids.

#### 2.2.2. Effect of Fungicides on Total Phenols, Total Antioxidant Content, and Antioxidant Capacity of Aqueous Extracts from Cucumber Seedlings

**Figure 5:**
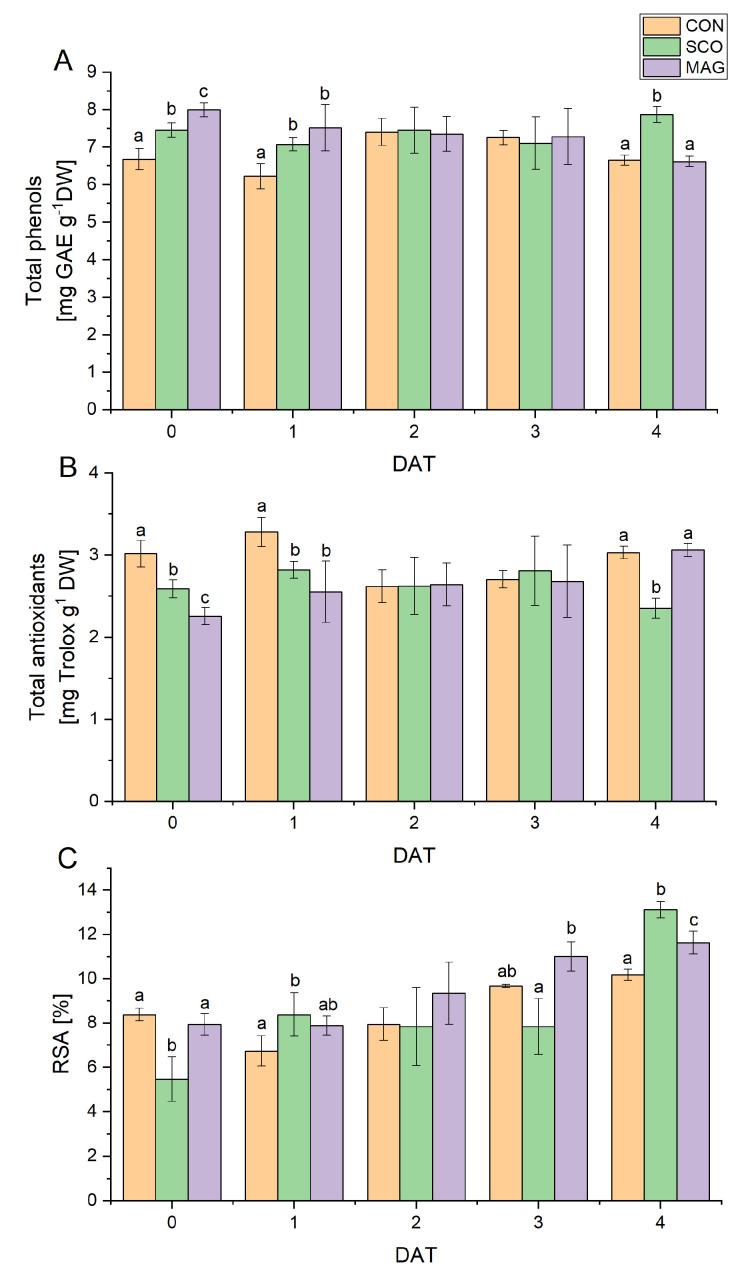
Content of polyphenolic compounds (**A**), total antioxidants (**B**), and antioxidant activity (**C**) in the extracts of aboveground parts of cucumber seedlings treated with tested pesticides (as foliar application) or untreated. Different letters represent values significantly different (*p* ≤ 0.05) between treatments for particular day of development of the germinating seeds.

The presence of secondary metabolites, such as polyphenols and antioxidants, can provide information about the plant’s defence mechanisms against stress conditions [15]. The total phenol (Figure 5A) and total antioxidant contents (Figure 5B) were in the range of 5.89–8.17 mg GAE g^−1^ DW and 2.15–3.10 mg Trolox g^−1^ DW, respectively. The lowest value of polyphenols was determined at 1 DAT for the control, while the highest was at 0 DAT for MAG. The lowest antioxidant content was at 0 DAT for MAG, and the highest antioxidant content was at 1 DAT for the control. For the control and SCO samples, these values fluctuated over time. The changes in the total phenols and antioxidant content in the MAG samples are inversely proportional. A linear decrease in phenolic compounds from 7.99 ± 0.18 to 6.62 ± 0.14 mg GAE g^−1^ DW was observed. However, the total antioxidant content increased from 2.26 ± 0.11 to 3.06 ± 0.08 mg Trolox g^−1^ DW. The antioxidant activity (Figure 5C), determined as the ability to scavenge free radicals, was in the range of 4.45–13.79%. The lowest value of RSA was determined for 0 DAT, and the highest for 4 DAT was for SCO. In the case of the control samples, a linear increase in antioxidant activity after 1 DAT from 6.73 ± 0.69% to 10.19 ± 0.25% was observed. A similar upward trend was observed for MAG, with a 1.44% higher final value compared to the control. On the other hand, in the case of SCO, the increase in antioxidant activity jumped from the lowest observed value of 5.46 ± 1.01% up to 13.12 ± 0.37%; however, from 1 DAT to 3 DAT, it remained at a level of approximately 8%.

#### 2.2.3. Effect of Scorpion and Magnicur Finito on Adenylate Content and AEC Ratio in Cucumber Seedlings

**Figure 6:**
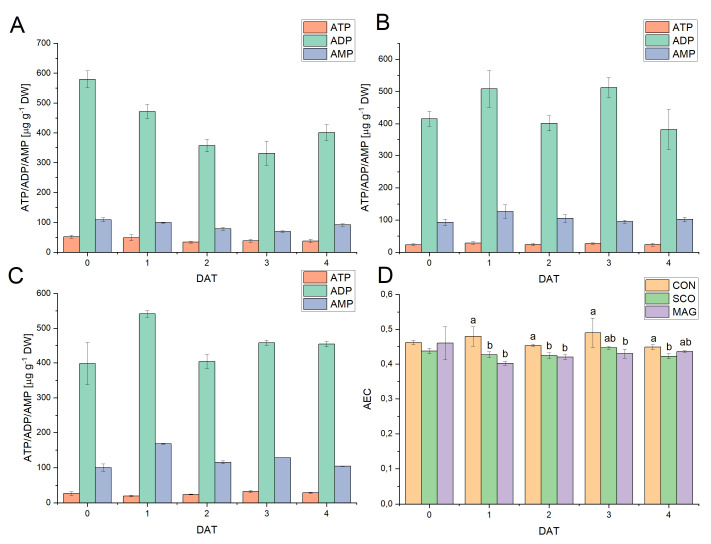
Energetic status of cucumber seedlings in the control (**A**) or treated (as foliar application) with the tested pesticides (SCO (**B**) and MAG (**C**)), presented as the contents of ATP, ADP, and AMP. Panel (**D**) shows the calculated adenylate energy charge (AEC) in the aboveground parts of cucumber seedlings (shoot includes hypocotyl, two green cotyledons, and true leaves). Different letters represent values significantly different (*p* ≤ 0.05) between treatments for a particular day of development of the germinating seeds.

Concerning the energetic aspects of metabolism in the studied cucumber plants, the content of three adenylates (ATP, ADP, AMP) and AEC ratio for the aboveground parts (Figure 6) and for roots (Table 3) were determined. The highest share of all adenylates was ADP, while the lowest was ATP for both the roots and aboveground parts. The ATP content in the aboveground parts remained constant and fell in the ranges of 31.05–57.39 µg g^−1^ DW for the controls, 20.59–29.28 µg g^−1^ DW for SCO, and 17.14–35.86 µg g^−1^ DW for MAG. Similar fluctuations were noted for the AMP for SCO, the content of which was at the level of 83.16–147.39. Significant differences in the AMP content were noticed between the control and MAG at 2, 3, and 4 DAT. The biggest changes were perceived in the content of ADP. For the control, there was a linear decrease from 579.77 ± 28.01 to 331.32 ± 40.45 at 3 DAT and then an increase again to 400.86 ± 27.34 at 4 DAT. However, this relationship did not occur in the case of SCO and MAG, which may suggest the effect of a fungicide on plant metabolism. To better define the metabolic changes taking place in the developing cucumber seedlings, the AEC ratio was determined. The AEC ratio was in the range of 0.39 to 0.53, which corresponds to the predominance of catabolic processes. Significant differences were noticed between the control and SCO groups, as well as between the control and MAG groups, which faded during the following days. However, these differences did not occur between the applied fungicides.

As with the aboveground part, ADP was the highest content among the adenylates, while AMP was the lowest. The AEC ratio was in the range of 0.40–0.49, with a higher AEC value recorded at 3 DAT. The initial decrease indicates strongly occurring catabolic processes, while the increase in the AEC parameter from 2 DAT suggests an increase in the share of anabolic processes.

#### 2.2.4. Effect of Fungicides on the^31^P NMR Profiles of Extracts of the Green Part of Cucumber Plants

**Figure 7:**
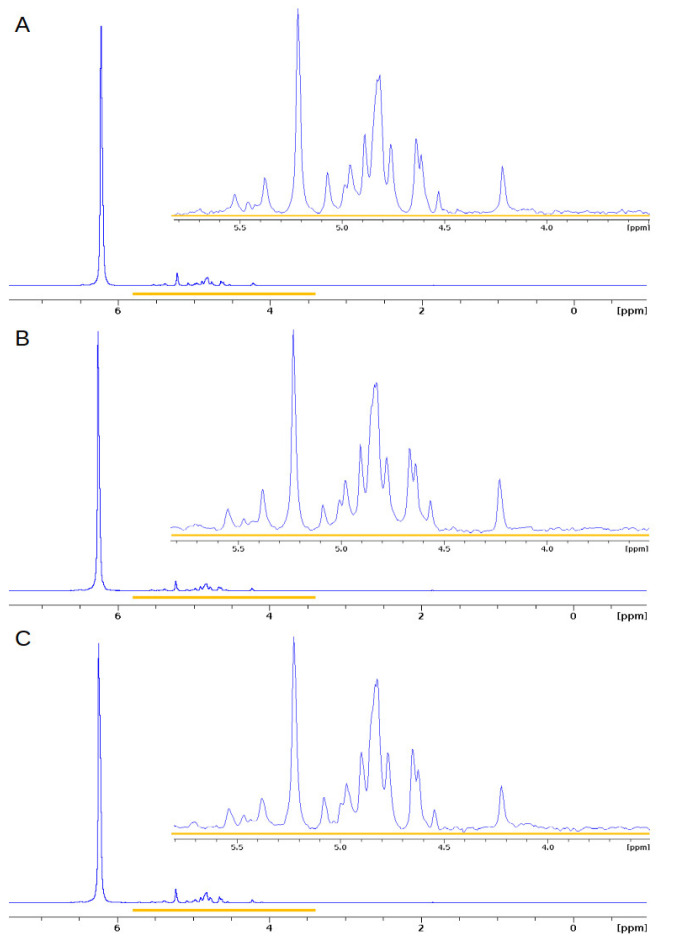
Normalised ^31^P NMR spectra of extracts of the foliar treatment of aboveground part of cucumber seedlings from 2 DAT of (**A**) CON, (**B**) SCO, and (**C**) MAG. In the right upper part of the panel, the enlarged part of the spectrum is presented to show subtle differences in its structure.

The percentage share of the phosphorus groups is presented in Table 4. Orthophosphates had the highest content of all phosphorus compounds, and their share ranged from 76.3 to 88.5%. However, the smallest group was phosphodiesters, the content of which did not exceed 1%. Figure 7 shows the compiled ^31^P NMR spectra of the samples at 2 DAT. Based on the obtained data, we conclude that the SCO and MAG profiles did not differ from the control with respect to the presence of various forms of phosphorus, and the observed differences are only quantitative.

## 3. Discussion

Fungal diseases of plants are one of the key factors affecting not only the size, but also the quality of crops, thus leading to serious economic losses. To minimise such losses, fungicides, as a broad group of various chemicals, are commonly used in the treatment of fungal diseases in fruits, vegetables, cereals, and other crop plants. However, there is a new trend in agriculture involving the use of certain groups of fungicides to improve the physiology of healthy plants to prevent against fungal infection [23,28]. This alternative usage mainly relates to strobilurins and carboxamides, which cause, inter alia, changes in the hormonal economy, ethylene reductions, or prolonged photosynthesis efficiency, thus leading to an increase in the productivity and quality of crops [27,28]. Studies on the positive effects of fungicides on plants have mainly concentrated on the photosynthesis process. Regardless of the new possibility of applying fungicides and the physiological benefits of plants treated with them, pesticides, including fungicides, are toxins by definition. Therefore, the application of fungicides to healthy plants may induce many concerns, and to date, only a few reports have indicated the toxic effect of fungicides on plant metabolism. Disruptions caused by fungicide application may lead to a reduction in growth [32], alterations in carbon and nitrogen metabolism [33], decreased nutrient availability [15], and changes in the development of reproductive organs [15]. Furthermore, commercially available fungicidal formulations usually consist of a mixture of at least two active substances, and there is little information on the influence of such preparations on plant metabolism. Therefore, the results of our two-variant study focusing on the possible effects of two fungicide preparations on selected aspects of plant metabolism may be treated as the next puzzle in this discussion.

### 3.1. Variant 1—Presowing Seed Treatment

Some fungicides, such as difenoconazole (active substances of SCO), are not only systemic foliar but also seed treatment agents [34]. Additionally, the literature data indicate that azoxystrobin is stable and its residues have been found in different matrices [35]. Furthermore, the application of these chemicals leads to an increase in long-term residues in food and in the environment [36]; therefore, they pose a threat to human health and nontarget organisms [34]. Because fungicidal residues might be deposited in seeds, and mildew disease may also be transferred by the seeds, the presowing seed application was tested.

First, the fungicides were applied to germinating seeds. SCO up to 4 DAT reduced the number of germinated seeds. The literature data indicated that azoxystrobin and difenoconazole transiently increase the abscisic acid (ABA) content [23,24,25]. Various studies have documented the negative effect of ABA on seed germination [37,38,39]. The high level of ABA inhibits seed germination and root elongation. However, these effects are temporary, and the percentage of germinated seeds at 7 DAT reaches a value comparable to that of the control.

The phosphorus present in plant seeds is stored in the form of phytate complexes of phytic acid with metal ions [40]. During the early stage of germination, phytase catalyses the enzymatic hydrolysis of phytic acid to inositol with the release of orthophosphate ions [41,42]. The slight increase in the content of phytic acid at 1 DAT is probably related to the release of metal ions from phytate. Phytic acid decreased by approximately 17% for the control, 14% for SCO, and 26% for MAG at 7 DAT compared to 0 DAT. Data from the literature indicate that during germination, the content of phytic acid decreases by 25–35% in sorghum, and these values depend on the variety [43]. In this respect, our study does not indicate a negative influence of the applied fungicide preparations on phytases, whose activity increases properly over time, up to 7 DAT in the case of the preparations used and 4 DAT in the case of the control. This may indicate a beneficial effect of SCO and MAG on phytases by prolonging their enzymatic activity. However, the trend of changes in phytic acid and Pi content does not correspond to changes in phytase activity. This may result from the fact that the phytases present in plant tissues are mainly classified as histidine acid phosphatases (HAPs) [44]. The data indicate that plant HAPs show broad substrate specificity and may catalyse not only the dephosphorylation of phytic acid, but also the hydrolysis of other phosphate esters, such as fructose 6-phosphate, B-glycerophosphate, Na-pyrophosphate, and adenylates (ATP, AMP) [45]. Currently, there is no information on the substrate specificity of phytases isolated from the germinating seeds of cucumber; nevertheless, the possible broad substrate specificity of this enzyme may also explain the two times lower ADP content in SCO compared to CON, where the phytase activity at 7 DAT was three times lower.

The literature data provide information that the ATP content increases during seed imbibition [46]. Such a dependence was not observed in our study. These kinds of differences follow from species and families of plant distinction, in which metabolic changes can occur in disparate ways. The highest content of adenylate was ADP, which indicates numerous metabolic changes with the use of ATP. Additionally, the AEC parameter value above 0.5 suggests that catabolic processes are not dominant [47], which confirms this conclusion. Simultaneously, the AEC remained constant until 4 DAT, and then a decrease was noticed. These results are consistent with the literature data [48]. During the process of germination, when the grain swells and metabolic processes are activated, reserve material is simultaneously decomposed, and numerous other compounds that are the building blocks of cell walls, DNA, or proteins are synthesised [49]. The decreasing value of the AEC parameter additionally indicates that the ratio of the share of anabolic and catabolic processes is changing. This is related to the fact that in germinating seeds, the respiration processes initially dominate, while when the cotyledons appear, the sprouts start to carry out photosynthesis. Differences in the biochemical substances occurring during the germination process were also observed as a result of the ^31^P NMR measurements. The most important were the changes in the relative amounts (%) of individual forms of phosphorus compounds and the appearance of additional forms of these substances. In addition, as a function of time, the relationship of individual signals coming from phosphomonoesters changed.

### 3.2. Variant 2—Foliar Application

Photosynthetic pigments are crucial in photosynthesis because they initiate the transfer of energy from absorbed light. In addition, their specific content may provide information about plant development and changes in metabolism. The decrease in chlorophyll concentration is often caused by environmental stress, such as drought [50] and salt stress [51]. The group of stressors also includes harmful chemicals, such as fungicides [15,52]. However, the data indicated that triazole and strobilurin fungicides may act as protectors by increasing tolerance to environmental stress [21]. To limit the intense increase in chlorophyll content and to intensify the susceptibility of cucumber seedlings to fungicides, the tested plants were watered without any nutrition from the beginning of the experiment. Our results show that despite the one-day decrease in SCO at 1 DAT, a higher chlorophyll content was observed on the last day of the experiment compared to the control. Data from the literature indicate that triazoles and strobilurins increase the chlorophyll content in cucumber plants [25,27]. At the same time, MAG did not increase the chlorophyll content, which is consistent with the literature data [53].

In the process of evolution, plants have developed various defence mechanisms including the synthesis of metabolites, such as phenolic compounds [54,55] as well as an increase in the activity of antioxidative enzymes [56], protecting against the negative effects of environmental stress. The higher content of antioxidants in the seedlings after treatment with MAG compared to the control may refer to the activation of lignin synthesis by propamocarb [20]. This activation takes place at the beginning of the phenylpropanoid pathway, which is confirmed by a correlated increase in antioxidant compounds with a decrease in the content of phenolic compounds. Additionally, phenolic compounds lose some of their antioxidant activity during plant growth as they become part of the structural framework of the growing plant [49].

The occurrence of significant differences in AEC values between SCO and the control as well as MAG and the control indicates that the tested fungicide preparations influenced the metabolism in the aboveground parts of the cucumber seedlings. In the case of roots, no such significant differences were noticed in the values of adenylated energy charge with respect to the control. The tested preparations, as a systemic agent, are distributed throughout the plant. The lack of differences in AEC may indicate that they do not disturb metabolic processes in the roots or that tested fungicides were transported and thus were only active within the aboveground parts, which are directly exposed to mildew infection. Because no negative changes were recorded in the root metabolism with respect to the AEC ratio, the plant can take up nutrients undisturbed, which seems to be an important advantage in the overall development of plants. Additionally, the obtained ^31^P NMR data indicate that the tested fungicides did not affect phosphorus metabolism in the aboveground parts. The differences between the individual groups of phosphorus compounds are only quantitative.

## 4. Materials and Methods

### 4.1. Plant Material and Experimental Design

The plant seeds of *Cucumis sativus* variety Izyd F1 were obtained from a local plant breeding and seed company (PNOS Sp. z o.o., Ożarów Mazowiecki, Poland) and stored at room temperature in their original packaging until use. The untreated seeds before use were surface-sterilised with 70% ethanol for 30 s and 0.5% (*v*/*v*) sodium hypochlorite solution for 15 min, drained, and rinsed with distilled water until they reached a neutral pH.

The effect of fungicides on cucumber growth was investigated separately for Scorpion 325 SC (Agrecol) and Magnicur Finito 687,5 SC (Protect Garden, Bayern). The final concentrations of applied fungicides were 0.1% and 0.3%, respectively, in accordance with the manufacturers’ recommendations. The active substances in Scorpion 325 SC are azoxystrobin (200 g L^−1^) and difenoconazole (125 g L^−1^), while those in MagnicurFinito 687,5 are propamocarb hydrochloride (55.31% *w*/*w*) and fluopicolide (62.5% *w*/*w*).

The experiments were performed in two variants, in which the effects of foliar application or incrustation of fungicides were examined. *Variant 1*—*presowing seed treatment* was conducted because mildew disease might be transmitted by seeds [10,57] and because the residue of active compounds of fungicides has been found in cucumber fruit [58,59]. As the chemicals were detected in the cucumber fruit and there is no information about the effect of fungicides on seed germination, presowing seed treatment was applied. *Variant 2*—*foliar application* corresponds to the regular application of the tested fungicides. As these fungicide preparations may be used for the prevention of mildew disease, the effect on seedlings was determined.

*Variant 1*—*presowing seed treatment*

A 1.0 ± 0.1 g sample of sterilised seed (corresponds to an average of 34–36 of seeds) was soaked in 5 mL of distilled water or tested fungicide solution for 4 h in darkness at room temperature. After that, the water or fungicide solution was discarded, and the seeds were placed in a Petri dish (Ø = 10 cm) containing two layers of filter paper moistened with distilled water. The samples were cultivated in phytotron FITO DOU (Biogenet, Poland) equipped with white (cold white (CW); warm white (WW)), blue (blue (B), λ = 460–480 nm; deep blue (DB), λ = 430–450 nm), and red (red (R), λ = 630–650 nm; deep red (DR), λ = 650–670 nm; far red (FR), λ = 710–740 nm) LEDs under dark (24 h darkness) and then under light conditions in a photoperiod regime (16 h/8 h light/dark, 25 °C/20 °C (day/night), with 70% air humidity). The LEDs were imitated sunlight conditions (the share and wavelength of individual sources of light were: 50% CW, 50% WW, 50% B, 50% DB, 50% R, 50% DR, and 50% FR). The plants were watered two times per day with 5 mL of distilled water. The experiment was conducted for five days (0 DAT was defined after 4 h of soaking). The germinating seeds were collected every day in five repetitions for each tested condition, and the number of germinated and non-germinated seeds was counted. The germination percentage was calculated as the number of sprouted seeds divided by the number of total seeds and expressed as a percentage for each day of plant development.

*Variant 2*—*foliar application*

One gram of sterilised seeds was imbibed in 5 mL of distilled water for 4 h in the dark at room temperature. The imbibed seeds were transferred to Petri dishes (Ø = 10 cm) filled with two layers of filter paper and moistened with 5 mL of distilled water twice a day. The plants were cultivated in the phytotron under greenhouse conditions (first 24 h in the dark, and then the next photoperiod was applied: 25/20 °C, day/night; humidity, 70%; light/dark, 16 h/8 h). On the eighth day of growth, the leaves of the cucumber seedlings were sprayed with 1 mL of the tested fungicide solution. The tested fungicides were applied to plants separately. After treatment, the seedlings were cultivated under the previous conditions until sampling. The seedlings (*n* = 10) were divided into aboveground parts and roots and harvested in five repetitions for individual experimental conditions by five days after treatment (DAT), starting two hours after spraying (0 DAT). 

The harvested samples were immediately frozen in liquid nitrogen and freeze-dried at −50 °C (freeze dryer ChristAlpha 1-2 LDplus, Osterode am Harz, Germany). The lyophilised samples were ground using a cryogenic mill machine (SPEX 6775 Freezer/Mill; SpexSamplePrep, Metuchen, NJ, USA). The cryogenic grinder was precooled for 5 min, and then samples were pulverised with a magnetically driven impactor at 15 s^−1^ in a liquid nitrogen bath. The cucumber material was subjected to two 30 s grinding cycles, with a 1 min intercool, which provided proper cryogenic temperatures for the samples at all grinding times. The samples were stored at −28 °C until further analysis. 

For *Variant 1*, the phytase activity, content of phytic acid, inorganic phosphates (Pi), proteins, adenosine monophosphate (AMP), adenosine diphosphate (ADP), adenosine triphosphate (ATP), and ^31^P NMR phosphorus profiles were determined. For *Variant 2*, the contents of photosynthetic pigments (chlorophyll ‘a’, chlorophyll ‘b’, and carotenoid), adenylates, phenolic compounds, antioxidant compounds, antioxidant capacity, and ^31^P NMR phosphorus profiles were determined. Determinations were performed in triplicate for each sample. All reagents used except malachite green and surfactant (CHAPS) were analytical grade and were purchased from Avantor Performance Materials Poland S.A. (Gliwice, Poland) and Merck (Merck Millipore, Darmstadt, Germany) and used without further purification. The water was treated in a Milli-Q water purification system (Millipore, Bedford, MA, USA). 

### 4.2. Determination of Protein Content and Acid Phytase Activity

The total protein content and acid phytase activity were determined according to Afify et al. [43] with some modifications. A crude enzyme extract was prepared by homogenising 250 mg of powdered plant material in ice-cold extraction buffer (10 mM Tris-HCl, pH 7.0, containing reduced glutathione, 0.5 mM). Solid cetylpyridinium bromide was added to the suspension (10 mg, final concentration 0.5% (*w*/*v*) and homogenised twice for 30 s, with a 15 s delay, using a Hilsher UP200HT ultrasonic homogeniser (26 kHz, 200 W, HilsherUltrasonics GmbH, Teltow, Germany). The crude extracts were centrifuged at 13,000× *g* for 30 min at 4 °C. The supernatant containing phytase activity was filtered using syringe filters (nylon membrane, pore size 0.45 μm) and used for the enzymatic assay.

The concentration of soluble protein in the extracts was determined according to the Bradford assay [60] with bovine serum albumin as the standard (y = 0.2802x + 0.3800, R^2^ = 0.9945).

The phytase activity was determined by measuring its ability to release inorganic phosphates (Pi). The reaction solution contained 230 µL of sodium acetate buffer (100 mM, pH 5.0, containing sodium phytate (1 mM) and CaCl_2_ (1 mM)), and 20 µL of the enzymatic extract was incubated at 37 °C for 1 h. The reaction was stopped by enzyme degradation using 50 µL of 50% TCA. The release Pi was determined by adding 700 µL of ammonium molybdate solution (1:6 solution of 10% (*w/v*) ascorbic acid and 0.45% (*w/v*) ammonium molybdate in 0.5 M H_2_SO_4_) and incubated at 37 °C for 10 min. The mixture was centrifuged at 13,000× *g* for 5 min to remove precipitated protein. The absorbance was measured at 820 nm against a background sample in which the enzyme was inactivated at the start of the reaction. The Pi concentration was calculated based on a calibration curve using KH_2_PO_4_ as a reference (y = 3.8222x − 0.0081, R^2^ = 0.9993). One unit of phytase activity (U) was defined as the amount of enzyme that releases 1 µmol of Pi from sodium phytate per minute under these conditions. 

### 4.3. Determination of Phytic Acid

Fifty milligrams of ground cucumber samples were extracted with 1.5 mL of 0.4 M HCl for 3.5 h on the platform shaker at 600 rotations per minute (Heidolph Vibramax 100, Schwabach, Germany). Then, the extracts were centrifuged at 13,000× *g* for 30 min and filtered using syringe filters (nylon membrane, pore size 0.45 μm). 

The phytic acid content in the extracts was determined by the method of Haug and Lantzsh [61] with minor modifications. Up to 0.5 mL of extract and 1 mL of ferric (III) solution (ammonium iron (III) sulphate · 12 H_2_O in 2 M HCl) were added. The mixture was heated (100 °C) for 30 min in a dry heating block (myBlock Mini Dry Bath; Benchmark, Sayreville, NJ, USA). After cooling at 4 °C for 30 min to precipitate the iron–phytic acid complex, the samples were centrifuged for 30 min at 13,000 rpm, and the supernatant was collected. Then, 750 µL of 2,2′-bipyridine solution (1% 2,2′-bipyridine and 1% thioglycolic acid in water) was added to 500 µL of the supernatant. The absorbance was measured at 519 nm against distilled water. The phytic acid concentration was calculated based on the calibration curve (y = −0.0036x + 0.9983, R^2^ = 0.9986) using sodium phytate as a reference. The amount of phytate in the tested sample was expressed as mg of phytate per gram of dry mass (mg g^−1^ DW).

### 4.4. Isolation of Phosphorus Compounds

Phosphorus compounds from the cucumber samples were isolated using ultrasound-assisted extraction (UAE) according to Wieczorek et al. [62], with some modifications. Approximately 1 g of sample was suspended in 5 mL of 35% perchloric acid solution and extracted in an ultrasonic cleaning bath (Cole-Parmer 8891, 42 kHz, 100 W; Vernon Hills, IL, USA) at 25 °C for 30 min. The whole extract was centrifuged at 6000× *g* for 5 min, the supernatant was collected, and another batch of 5 mL of perchloric acid was added to the plant material. Each sample was extracted three times. After extraction, the samples were washed with 5 mL of distilled water and centrifuged at 5000× *g* for 5 min. The supernatant was combined with the extracts and neutralised to pH 7.0 ± 0.5 with solid potassium hydroxide. The mixture was cooled at 4 °C for 30 min to precipitate potassium perchlorate solid and centrifuged at 5000× *g* for 5 min. The supernatant was diluted with distilled water to 25 mL, filtered using filter paper (Watchman No. 41), and divided into two parts. Two millilitres of extracts were frozen at −28 °C until the determination of Pi content, while the rest was lyophilised at −50 °C (ChristAlpha 1-2 Ldplus freeze dryer, Osterode am Harz, Germany) and stored at −28 °C until ^31^P NMR analysis.

### 4.5. Determination of Inorganic Phosphorus Content

The content of water-soluble inorganic phosphorus forms (Pi) was determined according to Forlani et al. [63], with some modifications. Up to 100 µL of each cucumber extract was added to 1.0 mL of the malachite green–molybdate acid solution. After exactly 60 s, the mixture was supplemented with 100 µL of 34% (*w*/*v*) sodium citrate solution. Subsequently, the absorbance of the coloured complex was measured at 660 nm against the water blank within 20 min of the addition of the colorimetric solution. The concentration of orthophosphate ions was calculated based on the calibration curve traced with KH_2_PO_4_ (y = 5.7761x + 0.0297, R^2^ = 0.9983). The amount of Pi was expressed as mg PO_4_^3−^ g^−1^ DW.

### 4.6. Determination of Phosphorus Profiles Using ^31^P NMR

^31^P NMR spectroscopy was carried out to define the phosphorus profiles of the seeds. Each freeze-dried extract obtained after seed extraction was redissolved in a mixture of 0.6 mL of deionised water and 0.4 mL of 0.1 M EDTA in 1 M NaOH. The samples were centrifuged at 13,000× *g* for 5 min to remove particles that might contribute to line broadening during NMR analysis. The pH of all of the obtained solutions was adjusted to 13.0 ± 0.5. The supernatant (500 μL) was transferred into a 5 mm NMR tube, together with a 1 mM solution of glufosinate as an internal reference standard (δ = 42.68 ppm). The ^31^P NMR experiments were performed using a 400 MHz Bruker Advance DRX spectrometer (Bruker, Rheinstetten, Germany) operating at a 161.98 MHz frequency. The data were acquired at 20 ± 1 °C using a 30° pulse, a 1.37 s acquisition time, and a 0.5 s relaxation delay. Broadband proton decoupling and a 20 Hz spin rate were used for all samples. The number of scans was 2048. The quantification of P species was performed by spectral deconvolution analysis based on chemical shifts and peak areas. The integration of peak areas was calculated on spectra processed with a line broadening of 2 Hz. The ^31^P NMR signal assignments were based on literature data [64,65]. The relative P concentrations in the neutralised extracts were estimated based on the total NMR signal area and presented as the percentage of each species using TopSpin version 3.6.2 software. All samples were prepared in triplicate for NMR analyses.

### 4.7. HPLC Analysis of ATP, ADP, and AMP as Nucleotides Involved in Energy Transformations at the Cellular Level

Adenine nucleotides (AMP, ADP, and ATP) were extracted from 100 mg of powdered plant material with 1.0 mL of ice-cold 6.0 M HClO_4_ and homogenised twice at 30 s with a 15 s delay using an ultrasonic homogeniser. Then, the mixture was centrifuged at 13,000× *g* at 4 °C for 10 min, and the supernatant was neutralised in a cold bath with 10.0 M KOH to pH 7.0. After re-centrifugation, the samples were filtered using syringe filters (nylon membrane, pore size 0.22 μm) and stored at −28 °C until high-performance liquid chromatography (HPLC) analysis.

The samples were analysed using a Dionex Ultimate^®^ 3000 HPLC system (Thermo Fisher Scientific, Waltham, Massachusetts, USA) equipped with a diode array detector (DAD-3000RS) that monitors the eluate at 254 nm. HPLC separations were performed using a mobile phase that consisted of a 5 mM KH_2_PO_4_ buffer (pH 7.0, containing 1% acetonitrile (ACN)) and ACN with an initial ratio of 100:0 (*v*/*v*). During the first 10 min, the composition of the mobile phase was unchanged, and the flow rate was 0.5 mL min^−1^. From 10 to 15 min, the contribution of phosphate buffer was reduced to 75%, which was accompanied by an increase in the flow rate to 1.0 mL min^−1^. Next, during the time from 20 to 25 min of analysis, the composition of the mobile phase was gradually returned to the initial parameters. These conditions were kept up to 30 min when the separation was finished. The samples (20 μL), maintained in an autosampler at 8 °C, were injected onto a 4.6 mm × 250 mm Phenomenex Gemini NX-C18 column connected to a dedicated SecurityGuard™ ULTRA Cartridge System, which was placed in a thermostat at a temperature of 30 °C. The external standards of ATM, ADP, and AMP (6.25–500 µM) were applied to quantify the adenylate contents and expressed as µg g^−1^ DW. The adenylate energy charge (AEC) was determined as AEC = ((ATP) + 0.5 (ADP))/((ATP) + (ADP) + (AMP)).

### 4.8. Determination of Photosynthetic Pigments

The contents of chlorophyll ‘a’, chlorophyll ‘b’, and carotenoids were determined spectrophotometrically according to Zlotek et al. [66], with minor modifications. A 50 mg sample was mixed with 25 mg of MgO to prevent the formation of pheophytin and extracted with 2 mL of 80% (*v*/*v*) acetone solution on a platform shaker at 600 rotations per minute (HeidolphVibramax 100, Schwabach, Germany) for 2 h under light protection. Then, the extracts were centrifuged at 13,000× *g* for 5 min, and the supernatant was collected. The absorbance of the extracts was measured against the extraction solution at 470, 645, and 663 nm for each sample using a UV–Vis spectrophotometer (Rayleigh UV2601 UV/VIS, Beijing, China). The total chlorophyll content was calculated using the following formula: chlorophyll = chlorophyll a + chlorophyll b = (12.72 A_663_ − 2.59 A_645_) + (22.88 A_645_ − 4.67 A_663_), while carotenoid = (1000 A_470_ − 3.27 × chlorophyll a − 104 × chlorophyll b)/229. The results are expressed as mg g^−1^ DW.

### 4.9. Determination of Total Phenolic and Antioxidant Compounds and Antioxidant Capacities

#### 4.9.1. Preparation of Aqueous Extract

One hundred milligrams of ground aboveground parts of the cucumber seedlings were extracted with 2 mL of distilled water for 1 h under dark conditions on a platform shaker at 600 rotations per minute (Heidolph Vibramax 100, Schwabach, Germany). After that, the extraction was carried out in an ultrasonic bath (Cole-Parmer 8891, 42 kHz, 100 W, Vernon Hills, USA) at room temperature for 20 min. Then, the extracts were filtered using syringe filters (nylon membrane, pore size 0.45 μm) and stored at −28 °C for further analysis.

#### 4.9.2. Total Phenol Content

The total content of phenolic compounds (TPCs) was determined according to Vale et al. [67], with some modifications. Fifty microliters of the aqueous extract were supplemented with 2.5 mL of Folin–Ciocalteu reagent (diluted 1/10), followed by 2 mL of Na_2_CO_3_ (7.5%, *w*/*v*). The mixture was kept at 45 °C for 15 min. After a predetermined time, the absorbance was measured at 765 nm using a UV–Vis spectrophotometer (Rayleigh UV2601 UV/VIS, Beijing, China). Gallic acid (GAE) was used as a reference to the standard curve (y = 0.0012 GA (µg mL^−1^) − 0.0182, R^2^ = 0.9982) and the TPC was expressed as mg gallic acid equivalents in g dried weight (mg GAE g^−1^ DW).

#### 4.9.3. Total Antioxidant Compound Content

The total content of antioxidant compounds was determined according to Korzeniowska et al. [68], with minor modifications. The ABTS working solution was used with Trolox as a positive control. The ABTS working solution was prepared by mixing 1 mL of ABTS (2,2′-azino-bis (3-ethylbenzothiazoline-6-sulphonic acid)) solution (14 mM) with 1 mL of a potassium persulfate solution (5 mM) and incubated overnight under dark conditions. After that, the mixture was appropriately diluted with water to obtain an absorbance of 0.750 ± 0.050 at 734 nm. Up to 100 µL of appropriately diluted aqueous extracts, 1 mL of ABTS working solution was added, and the absorbance was measured after 6 min at 734 nm against water. The absorbance of the extract without reagent was measured as the background of the sample. The total antioxidant content was calculated from the standard curve for methanolic Trolox solution (y = −0.0105x + 0.7047, R^2^ = 0.9988), and the results were expressed as mg Trolox g^−1^ DW.

#### 4.9.4. Antioxidant Activity Determination

The antioxidant capacities were measured by DPPH methods according to Korzeniowska et al. [68], with minor modifications. In the initial stage, a methanolic solution of DPPH (0.04 mg mL^−1^) was prepared and left for 30 min at room temperature under light protection. DPPH solution (1.95 mL) was added to 50 µL of extracts and incubated in darkness for 30 min. Then, the absorbance was measured at 517 nm against methanol. In addition, the background of the sample, which consisted of 50 µL of extract and 1.95 mL of methanol, was measured.

The antioxidant activity expressed as radical-scavenging activity (RSA) presents the discolouration of the DPPH and was calculated using the following formula: RSA (%) = {(A_control_ − (A_sample_ − A_background_))/A_control_]·100%, where A_control_ is the absorbance of DPPH solution, A_sample_ is the absorbance of the sample with DPPH solution, and A_background_ is the absorbance of the sample with methanol.

### 4.10. Statistical Analysis

Data are expressed as the mean ± SD of at least three repetitions of each treatment. All plots and statistical analyses were performed using OriginPro Version 2022 (OriginLab, Northampton, MA, USA). Data were analysed using one-way ANOVA and tested for significant (*p* ≤ 0.05) differences using Tukey’s test.

## 5. Conclusions

The beneficial changes caused by applying fungicides, known as the “green effect”, might suggest that these pesticides can be used as promoters of plant development, thus increasing crop yield and fruit quality [23]. However, the results of our study show that the character and range of metabolic changes caused by the tested systemic fungicides are not unambiguously positive. The application of fungicide formulation as a presowing seed treatment caused a perturbation in the phytase activity. As phytase plays a crucial role in the release of stored phosphorus, disturbances in the activity of these enzymes can lead to disorders in the energetic status of germinating seeds. In addition, the tested preparations changed the morphology of the germinating seeds, limiting the growth of the stem. Such changes are directly related to the plants’ response to stress [15]. Furthermore, the application of the tested fungicides to seedlings disrupted the energetic status and antioxidative system. Therefore, in our opinion, it is too early to unequivocally state the benefits of using systemic fungicides as preventive agents in crop protection. Moreover, such compounds may negatively affect other elements of the agrocenosis mycosphere, such as arbuscular mycorrhizal fungi. Additionally, the excessive use of pesticides will increase pathogen resistance to active substances, which, in time, will lead to a decrease in the number of effective fungicides.

## Figures and Tables

**Table 1:** Relative amounts (%) of the major P forms detected in alkalised extracts of germinating cucumber seeds. Chemical shifts of P forms are given in the table.

DAT	Treatments	P Forms (%)			
		Orthophosphates 5.5–7.0 ppm	Phosphomonoesters 3.0–5.5 ppm	Phospholipids 1.5–3.0 ppm	Other Phosphodiesters −2.0–1.5 ppm
0	CON	31.5	68.0	0.1	0.4
	SCO	28.0	71.6	0.2	0.2
	MAG	40.7	58.6	0.1	0.5
2	CON	24.5	75.3	0.1	0.0
	SCO	25.4	74.2	0.2	0.2
	MAG	36.5	62.1	0.4	1.0
4	CON	45.2	54.4	0.4	0.0
	SCO	28.0	71.6	0.4	0.0
	MAG	49.8	49.7	0.2	0.3

The standard error did not exceed 10% of the values given in the table.

**Table 2:** Content of photosynthetic pigments in aboveground part of cucumber seedlings.

Pigment	Treatments	Day after Treatment (DAT)				
		0	1	2	3	4
Chlorophyll ‘a’	CON	6.62 ± 0.56 a	6.68 ± 0.50 a	7.36 ± 0.74 a	6.41 ± 1.08 a	5.94 ± 0.70 a
	SCO	6.37 ± 1.05 a	5.69 ± 0.29 b	6.74 ± 0.80 a	6.68 ± 0.47 a	6.27 ± 0.27 a
	MAG	5.52 ± 0.24 a	6.38 ± 0.15 a	6.97 ± 0.20 a	6.23 ± 0.25 a	5.71 ± 0.35 a
Chlorophyll ‘b’	CON	2.23 ± 0.21 a	2.15 ± 0.21 ab	2.38 ± 0.28 a	2.26 ± 0.33 a	2.12 ± 0.35 a
	SCO	2.19 ± 0.38 a	2.09 ± 0.09 a	2.32 ± 0.31 a	2.39 ± 0.12 a	2.26 ± 0.04 a
	MAG	2.12 ± 0.12 a	2.36 ± 0.07 b	2.50 ± 0.20 a	2.44 ± 0.09 a	2.29 ± 0.18 a
Total chlorophyll	CON	8.85 ± 0.71 a	8.83 ± 0.66 a	9.74 ± 0.95 a	8.66 ± 1.31 a	8.06 ± 0.96 a
	SCO	8.56 ± 1.32 a	7.78 ± 0.35 b	9.06 ± 1.02 a	9.07 ± 0.54 a	8.52 ± 0.27 a
	MAG	7.74 ± 0.38 a	8.69 ± 0.22 a	9.46 ± 0.18 a	8.61 ± 0.32 a	8.02 ± 0.46 a
Carotenoids	CON	0.80 ± 0.09 a	0.74 ± 0.05 b	0.95 ± 0.12 a	0.69 ± 0.04 a	0.65 ± 0.08 a
	SCO	0.73 ± 0.12 a	0.61 ± 0.01 a	0.85 ± 0.12 a	0.66 ± 0.03 a	0.71 ± 0.02 a
	MAG	0.69 ± 0.04 a	0.65 ± 0.04 a	0.88 ± 0.04 a	0.65 ± 0.03 a	0.64 ± 0.02 a

Concentration of photosynthetic pigments is in mg g^−1^ DW. Different letters represent values significantly different at *p* ≤ 0.05 according to Tukey’s test.

**Table 3:** Content of adenylates (ATP, ADP, AMP) and AEC value in the extracts of cucumber roots of seedlings treated with pesticides or untreated.

Nucleotide Parameter	Treatments	Day after Treatment (DAT)				
		0	1	2	3	4
ATP (µg g^−1^ DW)	CON	47.84 ± 1.87 a	33.52 ± 1.82 a	96.32 ± 5.11 a	105.00 ± 15.44 ab	59.14 ± 3.81 a
	SCO	66.33 ± 3.61 ab	66.74 ± 6.68 b	85.57 ± 8.21 a	83.18 ± 10.54 a	75.17 ± 5.95 ab
	MAG	89.60 ± 10.42 b	43.83 ± 5.96 a	92.26 ± 9.84 a	111.39 ± 10.74 b	86.93 ± 15.83 b
ADP (µg g^−1^ DW)	CON	824.21 ± 52.79 a	463.19 ± 8.05 a	659.40 ± 38.99 a	731.60 ± 74.69 a	569.53 ± 89.44 a
	SCO	683.00 ± 23.08 a	723.64 ± 20.08 b	619.72 ± 78.64 a	596.40 ± 17.26 b	577.02 ± 42.67 a
	MAG	809.72 ± 79.41 a	605.89 ± 9.92 c	685.42 ± 52.98 a	693.15 ± 45.94 ab	580.01 ± 60.60 a
AMP (µg g^−1^ DW)	CON	207.40 ± 12.23 a	136.97 ± 2.84 a	156.23 ± 5.88 a	152.27 ± 20.92 a	128.42 ± 19.46 a
	SCO	183.70 ± 8.41 b	187.81 ± 1.32 b	141.04 ± 18.15 b	119.49 ± 5.44 b	137.50 ± 14.02 a
	MAG	212.40 ± 15.11 ab	164.54 ± 4.01 c	186.31 ± 16.29 ab	168.44 ± 18.85 ab	145.79 ± 15.59 a
AEC	CON	0.42 ± 0.02 a	0.42 ± 0.02 a	0.42 ± 0.02 a	0.42 ± 0.02 a	0.42 ± 0.02 a
	SCO	0.42 ± 0.00 a	0.42 ± 0.00 a	0.42 ± 0.00 a	0.42 ± 0.00 a	0.42 ± 0.00 a
	MAG	0.47 ± 0.01 a	0.47 ± 0.01 a	0.47 ± 0.01 a	0.47 ± 0.01 a	0.47 ± 0.01 a

Different letters represent values significantly different at *p* ≤ 0.05 according to Tukey’s test.

**Table 4:** Relative amounts (%) of the major P forms detected in alkalised extracts of green parts of cucumber seedlings. Chemical shifts of P forms are given in the table.

DAT	Treatments	P Forms (%)			
		Orthophosphates 5.5–7.0 ppm	Phosphomonoesters 3.0–5.5 ppm	Phospholipids 1.5–3.0 ppm	Other Phosphodiesters −2.0–1.5 ppm
0	CON	76.3	22.8	0.2	0.7
	SCO	86.1	13.3	0.1	0.4
	MAG	84.6	15.0	0.0	0.4
2	CON	84.5	15.4	0.1	0.0
	SCO	85.9	14.0	0.1	0.0
	MAG	82.6	16.9	0.1	0.4
4	CON	85.2	14.5	0.0	0.3
	SCO	88.5	11.1	0.1	0.3
	MAG	77.0	22.2	0.3	0.5

Standard error did not exceed 10% of the values given in the table.

## Data Availability

On request to those interested.
